# Perioperative complications of percutaneous nephrolithotomy and combined percutaneous nephrolithotomy with uni‑ and bilateral ureterorenoscopic stone therapy: results of the GRAND study

**DOI:** 10.1186/s12894-026-02357-1

**Published:** 2026-09-15

**Authors:** Benedikt Ebner, Paulo L. Pfitzinger, Troya Georgieva, Iulia Blajan, Moritz Happe, Yannic Volz, Maria Apfelbeck, Christian G. Stief, Michael Chaloupka, Nikolaos Pyrgidis

**Affiliations:** Department of Urology, University Hospital, Ludwig-Maximilians-University Munich, Marchioninistraße 15, Munich, 81377 Germany

**Keywords:** Ureteroscopy, Percutaneous nephrolithotomy, Urolithiasis, Perioperative outcomes

## Abstract

**Background:**

Percutaneous nephrolithotomy (PCNL) is a standard therapy for large renal stones. When combined with unilateral or bilateral ureterorenoscopy, stone-free rates may increase, but data on perioperative complications remain limited.

**Methods:**

We used the GeRmAn Nationwide inpatient Data (GRAND), provided by the Research Data Center of the Federal Bureau of Statistics (2005–2023). We assessed, through multiple patient-level analyses, perioperative morbidity, mortality, length of hospital stay, intensive care unit admissions, and the evolution of PCNL during the last years in Germany.

**Results:**

A total of 110,276 patients underwent PCNL (*n* = 98,965; 90%) and 11,208 patients (10%) underwent PCNL + unilateral ureterorenoscopy, respectively. The number of patients undergoing PCNL + bilateral ureterorenoscopy was very low (*n* = 103; 0.1%). Overall mortality was low after PCNL (0.2%) and PCNL + unilateral ureterorenoscopy (0.2%). PCNL + unilateral ureterorenoscopy was associated with increased odds of transfusion (3.3% versus 2.4%, OR: 1.3, *p* < 0.001), sepsis (2% versus 1.3%, OR: 1.6, *p* < 0.001), and acute kidney disease (2.6% versus 1.5%, OR: 1.6, *p* < 0.001) compared to PCNL. ICU admission rates were 1.9% after PCNL and 2.3% after PCNL + unilateral ureterorenoscopy (OR: 1.2, *p* = 0.004). Median length of hospital stay was 6 and 7 days, respectively.

**Conclusions:**

PCNL in Germany over the past two decades was associated with low perioperative morbidity and mortality. While PCNL + unilateral ureterorenoscopy is increasingly performed and is generally safe, it carries a modestly elevated risk profile. PCNL + bilateral ureterorenoscopy, in contrast, remains rarely performed. Prospective collaborative studies are warranted to define the optimal stone management strategies.

## Introduction

Percutaneous nephrolithotomy (PCNL) is the gold standard for the treatment of large renal stones, especially for those located in the lower calyx group [1]. When combined with a concurrent unilateral ureterorenoscopy as part of an endoscopic combined intrarenal surgery (ECIRS), it is possible to decrease the number of necessary punctures and increase the stone-free rate [2]. Studies have also shown that a combination with bilateral ureterorenoscopy within a simultaneous bilateral endoscopic surgery (SBES) can achieve complete stone clearance in both pelvicalyceal systems in selected cases [3].

Studies have demonstrated that miniaturizing the PCNL armamentarium or employing vacuum‑assisted systems can lower the complication rate [4, 5]. However, compared with other therapeutic modalities for urolithiasis, such as extracorporeal shock wave lithotripsy (ESWL) or ureterorenoscopy alone, PCNL may carry a higher risk of complications [6, 7]. In particular, postoperative fever, sepsis, bleeding requiring transfusion, and urinoma may occur often after PCNL [8, 9]. Nevertheless, comprehensive epidemiological studies focusing on the complication risk of PCNL alone, as well as in combination with unilateral or bilateral ureterorenoscopy, remain scarce.

In this context, we present, to the best of our knowledge, the largest study on this topic to date, assessing the current trends and major perioperative complications of PCNL alone, as well as with concomitant same unilateral or bilateral ureterorenoscopy in Germany from 2005 until 2023.

## Methods

### GeRmAn Nationwide inpatient Data (GRAND)

This study utilized nationwide inpatient hospital data from Germany spanning the years 2005 to 2023. The dataset encompasses information on patient comorbidities, surgical procedures, and perioperative outcomes from all hospitals across Germany, with the exception of psychiatric, forensic, and military institutions. These anonymized data are maintained by the German Office of Statistics (Wiesbaden, Germany) and were made available for analysis under an approved agreement (LMU − 4710 − 2022). Following the establishment of the diagnosis- and procedure-related remuneration system (G-DRG) in 2004, all hospitals in Germany are mandated to report inpatient treatment outcomes to the Institute for the Hospital Remuneration System for reimbursement purposes. Comorbidities and perioperative outcomes are documented using the International Statistical Classification of Diseases and Related Health Problems, 10th revision, German Modification (ICD-10-GM), while surgical procedures are coded according to the German Procedure Classification (OPS). The German Institute for Medical Documentation and Information provides coding guidelines to standardize data documentation across hospitals.

### Study population and outcomes

The study included patients who underwent unilateral PCNL (OPS codes: 5-550.20, 5-550.30, 5-562.7 or 5-562.6), PCNL + unilateral ureterorenoscopy (unilateral PCNL OPS codes plus concomitant unilateral ureterorenoscopy OPS codes), and PCNL + bilateral ureterorenoscopy (unilateral PCNL OPS codes plus concomitant bilateral ureterorenoscopy OPS codes) due to urolithiasis (ICD-10 code: N20) based on our prior publication on ureterorenoscopy [10]. To capture baseline characteristics and perioperative complications, additional ICD-10 and OPS codes were utilized. The primary objective of the analysis was to evaluate the impact of PCNL, PCNL with concomitant unilateral ureterorenoscopy, and PCNL with concomitant bilateral ureterorenoscopy on major perioperative outcomes during the hospital stay, including mortality, sepsis, acute kidney injury, blood transfusion, intensive care unit (ICU) admission, and length of hospital stay. Secondary outcomes examined differences in the patient baseline characteristics and trends in the utilization of these surgical approaches over time in Germany.

### Statistical analysis

All statistical analyses were conducted at the Research Data Center of the German Office of Statistics using R scripts developed by the research team (source: Research Data Center of the German Office of Statistics, DRG Statistics 2005–2023, own calculations). Since only aggregated results were accessible to the research team and individual patient data remained confidential, ethical approval and patient consent were not required under German regulations. Categorical variables were reported as absolute numbers and percentages, while continuous variables were presented as median values with interquartile range (IQR). Multivariable logistic and linear regression analyses were conducted to assess the influence of surgical approach (PCNL versus PCNL + unilateral ureterorenoscopy versus PCNL + bilateral ureterorenoscopy) on perioperative outcomes, including morbidity, mortality, hospital length of stay, and ICU admission. Adjustments were made for patient sex, age, obesity, history of chronic kidney disease, diabetes, and prior ureteral stenting. Results were expressed as odds ratios (ORs) with 95% confidence intervals (CIs), and a two-sided p-value of less than 0.05 was considered indicative of statistical significance.

## Results

### Baseline characteristics and trends

**Fig. 1:**
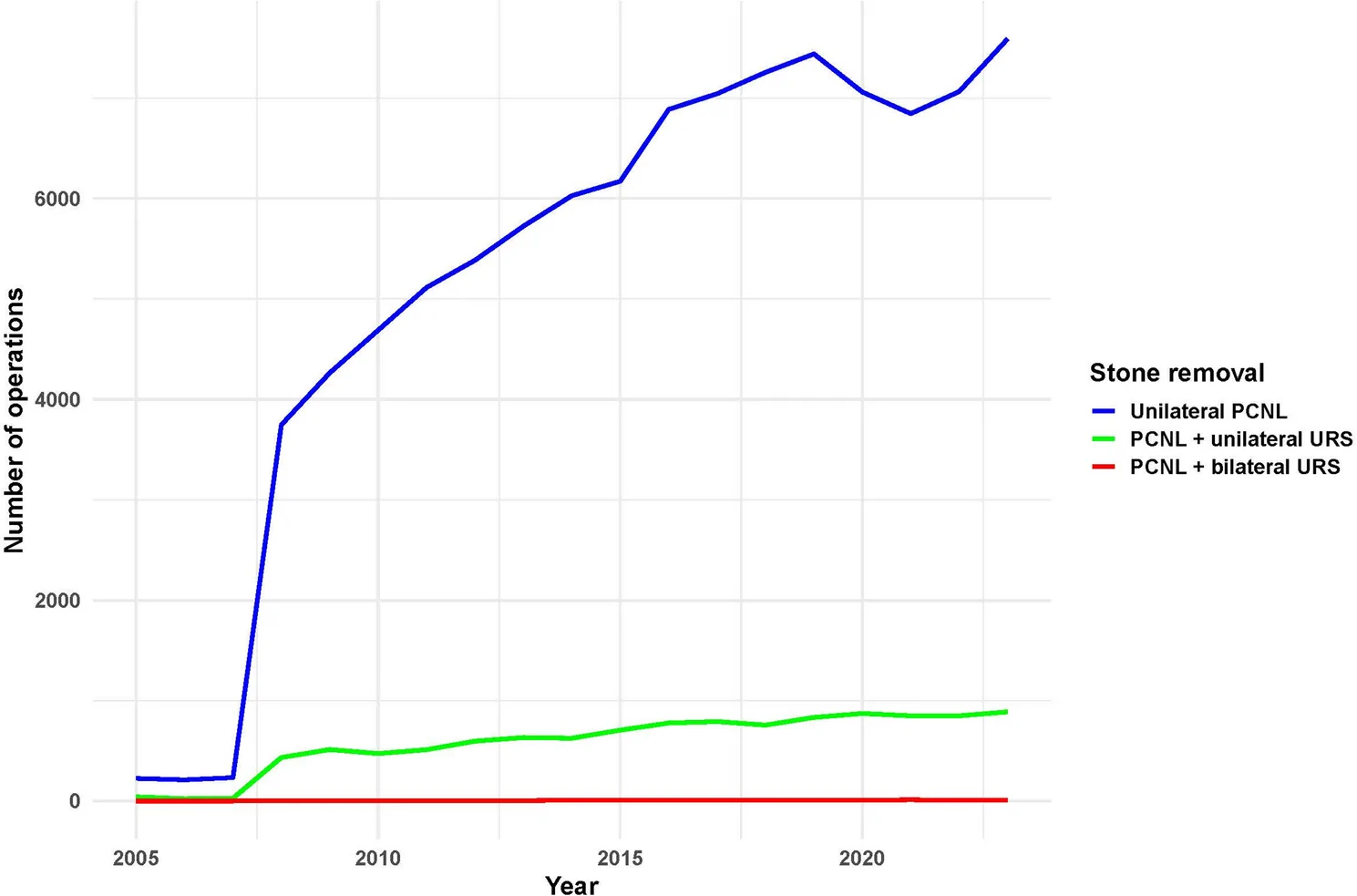
The annual trends of PCNL, PCNL + unilateral ureterorenoscopy, and PCNL + bilateral ureterorenoscopy in Germany for patients treated for urolithiasis. PCNL: percutaneous nephrolithotomy

We included 110,276 patients who underwent surgical stone removal via unilateral PCNL (*n* = 98,965, 90%), PCNL + unilateral ureterorenoscopy (*n* = 11,208, 10%), or PCNL + bilateral ureterorenoscopy (*n* = 103, 0.1%) between 2005 and 2023 in Germany. The median age of the overall cohort was 57 years (IQR: 46–67), and 59% were male. Among all patients, 9.5% were obese, 39% had hypertension, 17% had diabetes, and 6.7% had a history of chronic kidney disease. Prior ureteral stenting was performed in 40% of the total cohort. Compared to patients undergoing unilateral PCNL, those treated with PCNL + unilateral ureterorenoscopy or PCNL + bilateral ureterorenoscopy presented with higher rates of prior stenting (56% and 69%, respectively) and chronic kidney disease (8.5% and 16%, respectively) (Table 1). Notably, the utilization of PCNL and PCNL with unilateral ureterorenoscopy steadily increased between 2005 and 2023, followed by a decline during the COVID-19 pandemic. Notably, the adoption of PCNL with concomitant unilateral ureterorenoscopy has slightly risen in recent years, while PCNL with concomitant bilateral ureterorenoscopy is rarely performed in Germany (Fig. 1).

**Table 1:** Baseline characteristics of the included patients based on the surgical approach for stone management. Variables are presented as median with interquartile range or frequencies with proportions. ICU: intensive care unit, PCNL: percutaneous nephrolithotomy

Characteristic	Overall, *n* = 110,276	Unilateral PCNL, *n* = 98,965	PCNL + unilateral ureterorenoscopy, *n* = 11,208	PCNL + bilateral ureterorenoscopy, *n* = 103
Males	65,203 (59%)	58,394 (59%)	6,742 (60%)	67 (65%)
Age (years)	57 (46–67)	57 (46–67)	57 (45–67)	61 (48–70)
Obesity	10,492 (9.5%)	9,440 (9.5%)	1,040 (9.3%)	12 (12%)
Hypertension	43,406 (39%)	39,015 (39%)	4,353 (39%)	38 (37%)
Diabetes	19,177 (17%)	17,243 (17%)	1,916 (17%)	18 (17%)
Chronic kidney disease	7,387 (6.7%)	6,416 (6.5%)	955 (8.5%)	16 (16%)
Chronic heart failure	2,132 (1.9%)	1,875 (1.9%)	256 (2.3%)	1 (1.0%)
Prior stenting	44,638 (40%)	38,304 (39%)	6,263 (56%)	71 (69%)
Age group				
< 30	6,119 (5.5%)	5,397 (5.5%)	712 (6.4%)	10 (9.7%)
30–39	10,390 (9.4%)	9,271 (9.4%)	1,112 (9.9%)	7 (6.8%)
40–49	16,867 (15%)	15,156 (15%)	1,702 (15%)	9 (8.7%)
50–59	26,490 (24%)	23,916 (24%)	2,552 (23%)	22 (21%)
60–69	25,665 (23%)	23,116 (23%)	2,527 (23%)	22 (21%)
70–79	18,153 (16%)	16,265 (16%)	1,866 (17%)	22 (21%)
> 80	6,592 (6.0%)	5,844 (5.9%)	737 (6.6%)	11 (11%)

### Mortality, intensive care unit admission, and hospital stay

In terms of mortality, 207 deaths (0.2%) occurred after PCNL and 21 (0.2%) after PCNL + unilateral ureterorenoscopy, with no statistically significant difference between these groups (OR: 0.9, 95% CI: 0.54–1.34, *p* = 0.6). However, PCNL + bilateral ureterorenoscopy was associated with a significantly increased mortality rate of 1.9% (OR: 8, 95% CI: 1.3–26, *p* = 0.004) compared to unilateral PCNL. ICU admissions were higher both after PCNL + unilateral ureterorenoscopy (2.3% versus 1.9%, OR: 1.2, 95% CI: 1.1–1.4, *p* = 0.004) and after PCNL + bilateral ureterorenoscopy (5.8% versus 1.9, OR: 2.9, 95% CI: 1.1–6.2, *p* = 0.012). Moreover, the median length of hospital stay was 6 days (IQR: 4–8) after PCNL, 7 days (IQR: 4–11) after PCNL + unilateral ureterorenoscopy, and 10 days (IQR: 6–14) after PCNL + bilateral ureterorenoscopy. After adjustment for relevant confounders, PCNL + unilateral ureterorenoscopy was associated with a 1.8-day longer hospital stay (95% CI: 1.7–1.9, *p* < 0.001), while PCNL + bilateral ureterorenoscopy prolonged hospitalization by 4 days (95% CI: 3–6, *p* < 0.001) compared to PCNL (Table 2).

**Table 2:** Multivariable linear and logistic regression analysis for the effect of the surgical approach for stone management on major perioperative outcomes. All models are adjusted for sex, age, obesity, history of chronic kidney disease, diabetes, and prior stenting. The bold cells indicate statistically significant p-values. CI: confidence interval, ICU: intensive care unit, OR: odds ratio, PCNL: percutaneous nephrolithotomy

Outcome	PCNL		PCNL + unilateral ureterorenoscopy		PCNL + bilateral ureterorenoscopy	
	Cases	Estimate (95% CI), *p*-value	Cases	Estimate (95% CI), *p*-value	Cases	Estimate (95% CI), *p*-value
Mortality	207 (0.2%)	—	21 (0.2%)	0.9 (0.54, 1.34), 0.6	2 (1.9%)	8 (1.3, 26), 0.004
ICU admission	1,906 (1.9%)	—	262 (2.3%)	1.2 (1.1, 1.4), 0.004	6 (5.8%)	2.9 (1.1, 6.2), 0.012
Length of hospital stay	6 (4–8)	—	7 (4–11)	1.8 (1.7, 1.9), < 0.001	10 (6–14)	4 (3, 6), < 0.001
Transfusion	2,392 (2.4%)	—	373 (3.3%)	1.3 (1.2, 1.4), < 0.001	9 (8.7%)	3.2 (1.4, 6.1), < 0.001
Sepsis	1,238 (1.3%)	—	229 (2.0%)	1.6 (1.4, 1.8), < 0.001	5 (4.9%)	3.5 (1.2, 7.8), 0.007
Acute kidney disease	1,531 (1.5%)	—	296 (2.6%)	1.6 (1.4, 1.8), < 0.001	8 (7.8%)	4 (1.8, 8), < 0.001

### Perioperative morbidity

Perioperative morbidity was relatively low across all groups. However, compared to unilateral PCNL alone, both PCNL + unilateral ureterorenoscopy and PCNL + bilateral ureterorenoscopy were associated with significantly higher odds of major postoperative complications. Specifically, PCNL + unilateral ureterorenoscopy was associated with increased odds of transfusion (3.3% versus 2.4%, OR: 1.3, 95% CI: 1.2–1.4, *p* < 0.001), sepsis (2% versus 1.3%, OR: 1.6, 95% CI: 1.4–1.8, *p* < 0.001), and acute kidney disease (2.6% versus 1.5%, OR: 1.6, 95% CI: 1.4–1.8, *p* < 0.001). Accordingly, PCNL + bilateral ureterorenoscopy, though representing a small subset of the cohort, showed the highest rates of adverse events. In particular, compared to PCNL, PCNL + bilateral ureterorenoscopy was associated with increased odds of transfusion (8.7% versus 2.4%, OR: 3.2, 95% CI: 1.4–6.1, *p* < 0.001), sepsis (4.9% versus 1.3%, OR: 3.5, 95% CI: 1.2–7.8, *p* = 0.007), and acute kidney disease (7.8% versus 1.5%, OR: 4, 95% CI: 1.8–8, *p* < 0.001) (Table 2).

## Discussion

The present high-volume, nationwide analysis over the past two decades in Germany indicates an overall low complication rate of PCNL. Although combining the procedure with unilateral ureterorenoscopy increases the potential for complications, the overall level of risk remains low. So far, the combination of PCNL with bilateral ureterorenoscopy has been rarely performed and is associated with a troubling risk profile. However, due to the low number of cases, these results should be considered exploratory. In the multivariable analysis, we demonstrated that PCNL + unilateral ureterorenoscopy and particularly PCNL + bilateral ureterorenoscopy result in prolonged hospital stay, as well as in significantly increased rates of postoperative sepsis, acute kidney injury, transfusion, and ICU admission. Yet, the absolute increases in risk after combined surgery were modest. These real-world findings from the German inpatient population between 2005 and 2023 indicate that augmenting PCNL with a ureterorenoscopy increases the risk profile overall, and its necessity should therefore be carefully weighed in each clinical case.

The overall mortality of an endoscopic stone procedure is negligible in the context of ureterorenoscopy; previous studies from our group comprising 833,609 patients from 2005 to 2021 in Germany have shown that it was < 0.1% [10]. According to current evidence, the probability of death associated with PCNL also remains low. In a U.S. nationwide analysis of 80,097 patients from 1999 to 2009, Ghani et al. reported an in‑hospital mortality rate of up to 0.4% [11]. Similarly, Armitage et al. found a 30‑day in‑hospital mortality rate of 0.2% in a cohort of 5,750 PCNL procedures in England [12]. Our large‑data epidemiological study of all PCNLs performed in Germany, comprising 98,965 patients over the past 22 years, shows a mortality of 0.2%, placing it within the range reported by these studies. Risk factors such as age and comorbidity can, however, markedly increase PCNL‑related mortality. In multivariate analysis, Ghani et al. identified advanced age as a significant risk factor (OR 1.06; *p* = 0.008) [11]. In a retrospective analysis of 502 patients with chronic kidney disease, Srivastava et al. reported a mortality rate of 3.6% [13]. It should be noted, however, that patients undergoing PCNL generally carry a higher comorbidity burden than those treated by ureterorenoscopy. In our previous study, the prevalence of diabetes and chronic kidney disease among ureterorenoscopy patients was 6.9% and 4.3% [10], respectively, whereas our current study shows higher prevalences of 17% and 6.7% among PCNL patients. Our current study also reports a higher median age in the PCNL cohort—57 years versus 53 years for ureterorenoscopy patients. This clustering of risk factors may contribute to the comparatively higher mortality observed with PCNL relative to URS.

Systemic urinary tract infection is the most common complication after PCNL, and sepsis is the most common cause of death. Even with sterile urine preoperatively, the postoperative risk of sepsis remains substantial. A meta‑analysis by Zhou et al. reported postoperative sepsis rates between 2.4% and 14% in patients with a negative pre‑operative urine culture [9]. Interestingly, this high incidence was not evident in our epidemiological study. Despite the above‑average prevalence of known risk factors such as obesity (9.5%), diabetes (17%), and chronic renal failure (6.5%) [14], we observed a lower postoperative sepsis rate of 1.3%. The rate of ICU admissions (1.9%), however, proved to be higher compared to the current literature. In a retrospective multicenter study by Yaghoubian et al. comprising 320 patients, 0.9% met criteria for postoperative septic shock requiring ICU care [15]. This suggests that additional major complications in our series contributed to the increased ICU-admission rate. Postoperative bleeding and transfusion may be relevant factors. Yet the transfusion rate observed in our study (2.4%) was lower compared to international studies. In particular, a large U.S. database study reported a transfusion rate of 3.3% among 225,321 PCNL cases from 2003 to 2014 [16], and another nationwide US analysis of 80,097 patients (1999–2009) by Ghani et al. found a rate of 4% [11].

Since the concept of ECIRS was first described by Scoffone et al. in 2008 [17], numerous studies have reported advantages in stone‑free rates and complication profiles compared with conventional PCNL. A meta-analysis by Widyokirono et al. showed that the one-step stone-free rate of ECIRS was significantly higher compared with both the conventional PCNL (OR 5.14, *p* < 0.001) and mini PCNL (OR 4.27, *p* < 0.001). The risk of urosepsis after ECIRS was lower compared to conventional PCNL (OR 0.14, *p* = 0.02) [2]. Indeed, our study confirms a steady increase in the use of PCNL + unilateral ureterorenoscopy over the past decade. Nonetheless, we detected a rise in complication risk compared to conventional PCNL, although the overall level remained low. In multivariable analysis, PCNL + unilateral ureterorenoscopy was associated with higher odds of transfusion and sepsis compared with PCNL alone. Still, these increased odds after combined surgery were modest.

It should be noted that the observed increase in infectious and renal complications after PCNL + concomitant ureterorenoscopy may be attributed to specific technical aspects of the combined procedure. In many German centers, PCNL is still frequently performed in prone position using sequential Alken metal dilators rather than standard Amplatz access sheaths [18]. Furthermore, vacuum-assisted access systems are not routinely utilized [19]. Under these circumstances, the addition of flexible ureterorenoscopy may substantially increase irrigation flow while simultaneously limiting efficient outflow from the collecting system. This may result in significantly elevated intrarenal pelvic pressures during combined procedures [20]. Such elevated intrarenal pressures are known to promote pyelovenous and pyelolymphatic backflow and may therefore contribute directly to postoperative sepsis, systemic inflammatory complications, and acute kidney injury [20]. Consequently, the slightly increased complication rates observed in the PCNL + ureterorenoscopy cohorts may not necessarily reflect an inherent disadvantage of the ECIRS concept itself, but rather the hydrodynamic consequences of specific operative techniques still commonly employed in Germany. This issue is particularly important because several previous studies from high-volume ECIRS centers have demonstrated either comparable or even reduced infectious complications compared to conventional PCNL [21].

The concept of SBES was first described by Giusti et al. in 2018 and, in selected cases at an expert center, has yielded very good results [3, 22]. However, studies evaluating the complication rate of this technique are scarce. This is also reflected in our data: PCNL + bilateral ureterorenoscopy has been performed only rarely in Germany in recent years. Only 103 (0.09%) patients treated with PCNL during the study period underwent a concomitant bilateral ureterorenoscopy. Moreover, the complication profile is severe. In the multivariable analysis, the risk of urosepsis was significantly higher after PCNL + bilateral ureterorenoscopy compared to conventional PCNL. These findings suggest that PCNL + bilateral ureterorenoscopy should be reserved for selected centers and carefully chosen cases. Overall, the discrepancy between the lower complication rates reported in available studies and those seen in our real-world epidemiological data may be attributed to a selection bias, since available studies derive from high-volume centers with expertise [23].

Despite the large sample size and robust national coverage, our study is subject to several limitations inherent to administrative database analyses. The dataset is based on hospital coding for reimbursement, which may lead to inaccuracies or misclassification of diagnoses and procedures. Furthermore, key determinants of complication risk—such as stone burden, stone composition, stone-free rate, operative time, and surgeon experience—are not available. Similarly, specific information on the surgical armamentarium of PCNL, such as access sheath utilization, patient positioning, vacuum-assisted systems, or tract size, is also missing. Information on long-term follow-up, including readmission, reintervention, and outpatient complications, is also lacking. Moreover, we could not assess whether bilateral ureterorenoscopy was performed in the same session (simultaneously) or in a staged manner (separate procedures during the same hospital stay). Importantly, it could not be differentiated between mini-PCNL and PCNL. As the data are derived solely from the German healthcare system, the generalizability of our findings to other countries or healthcare systems may be limited. Finally, improvements in surgical technique and instrumentation over the nearly two-decade study period could have influenced the outcomes reported.

In this large nationwide analysis, PCNL demonstrated an overall favorable safety profile, with low perioperative morbidity and mortality. The combination of PCNL with unilateral ureterorenoscopy is increasingly adopted in Germany and, although associated with slightly higher complication rates, remains generally safe. On the contrary, PCNL + bilateral ureterorenoscopy has been used only in a very small number of patients and is so far associated with markedly higher risks, including mortality. Overall, given the important limitations of the present study, our findings should be considered primarily hypothesis-generating and do not reflect any causal relationship. Therefore, multicenter/multicounty prospective collaborative studies are warranted to define the optimal stone management strategies.

## Data Availability

The datasets generated and/or analyzed during the current study are not publicly available due to ethical and legal obligations.
